# A Large Thoracolumbosacral Meningomyelocele From Northern Tanzania: A Case Report

**DOI:** 10.1155/cris/5662565

**Published:** 2025-01-09

**Authors:** Mujaheed Suleman, Happiness Rabiel, Kerry Vaughan, Mathayo Shadrack, Goodluck Ndibalema, Raghav Lodhia, Jay Lodhia

**Affiliations:** ^1^Department of General Surgery, Kilimanjaro Christian Medical Centre, Moshi PO Box 3010, Tanzania; ^2^Department of Neurosurgery, Kilimanjaro Christian Medical Centre, Moshi PO Box 3310, Tanzania; ^3^Department of General Surgery, Faculty of Medicine, Kilimanjaro Christian Medical University College, Moshi PO Box 2240, Tanzania

**Keywords:** case report, folic acid, myelomeningocele, neural tube, obstetric ultrasound, spina bifida

## Abstract

Meningomyelocele and meningocele are types of neural tube defects, which are congenital abnormalities of the spine and spinal cord. These conditions are frequently encountered by pediatric neurosurgeons worldwide and represent a significant public health concern due to their association with a range of collateral conditions, other malformations, and increased morbidity. While many cases can be identified during prenatal ultrasound screenings, this is often challenging in resource-limited settings with poor health-seeking behaviors. Surgical intervention is the primary treatment for these defects, and while various methods are described in the literature, larger defects require complex flaps and techniques, with limited options available. Beyond early surgical intervention, patients require lifelong care involving multidisciplinary medical teams.

## 1. Introduction

Neural tube defects are the second most common congenital abnormalities preceded by heart diseases [1]. Also known as spinal dysraphia, these are congenital abnormalities resulting from the failure of neural tube closure during embryonic development. There are two dominant types of spina bifida including spina bifida occulta and spina bifida asperta which include exposure of the spinal cord to the environment [2]. Currently, myelomeningocele (MMC), the most common form of spina bifida is among the 10 leading causes of neonatal mortality associated with significant morbidity including effects of hydrocephalus, lower limb paralysis, and incontinence [1]. The global prevalence of neural tube defects is estimated at 4 per 1000 live births; however, large spinal defects are rare [3]. In this case report, we present a case of an unusually large thoracolumbosacral spina bifida that was successfully managed through simultaneous closure, skin grafting, and ventriculoperitoneal shunting.

## 2. Patient Information

A 3-day-old male baby was admitted to our pediatric neurosurgery ward, born via cesarean section at 39 weeks of gestation, scored an APGAR of 9 and 10 in the first and fifth minutes, respectively, and weighed 4000 g. He presented with a large back swelling since birth with a history of excessive crying associated with vomiting and history of serous discharge from the back swelling. There were no fevers or seizures reported. The mother also reported that her baby had an abnormally large head size. The mother had a late prenatal booking at 19 weeks gestation and tested negative for HIV, VDRL, toxoplasmosis, and CMV. The mother's age was 30 years, and she had one other child who was well. She started iron and folate supplements when she started prenatal clinic. She reported the pregnancy to be uneventful including blood pressure and glucose monitoring in desirable ranges.

### 2.1. Clinical Findings

**Figure 1:**
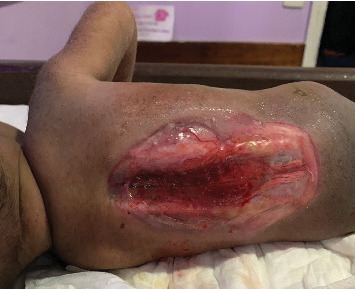
Clinical photograph showing large (thoracosacral) meningomyelocele with exposed lumbosacral nerve roots.

On examination, the baby was alert, irritable, with micrognathia, not pale, not dyspneic, not jaundiced, with an occipital-frontal circumference of 45 cm (above 97th percentile) and cranial sutural diastasis with bulging nontense fontanelles, sunset eyes, and distended scalp veins. The baby had an axillary temperature of 36.9°C, pulse rate of 135 bpm, and was saturating at 97% on room air. On examination of the spine, there was a large spinal defect ranging from the thoracic spine to the sacral region measuring 18 × 15 cm, exposing the lumbar and sacral nerve roots and plexus, with no spinous processes (Figure 1), no movement nor plantar reflexes in the lower extremities and had an atonic anal sphincter.

### 2.2. Timeline

The baby was born via cesarean section at a peripheral health center and was promptly referred to our tertiary care facility for further management. After thorough investigations, the baby underwent successful surgery to close the large meningomyelocele, along with the placement of a ventriculoperitoneal shunt. The recovery was smooth, and the child is now under regular follow-up with a multidisciplinary team.

### 2.3. Diagnostic Assessment

Laboratory values revealed a normal leucocyte count of 8.67 × 10^9^/L, hemoglobin of 15.7 g/dL (normocytic normochromic), and a normal platelet count of 161 × 10^9^/L. His serum creatinine was 97 µmol/L, urea of 6.04 mmol/L, potassium of 4.8 mmol/L, and sodium of 135.8 mmol/L. Brain ultrasound revealed a grossly dilated ventricular system with a thin peripheral rim of cortical tissue and dangling choroid with no falx, with features suggestive of holoprosencephaly. Echocardiogram was done and there were no cardiac anomalies.

**Figure 2:**
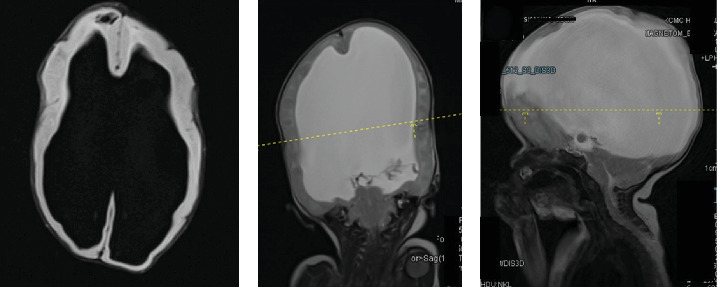
MRI scan of the brain revealed severe dilatation of the lateral ventricles, with thinning of brain parenchyma (A), the third and fourth ventricles appeared normal with effacement of aqueduct of Sylvius and tectal beaking, absent corpus callosum, small posterior fossa, and obliteration of cisterna magna. Bilateral clefts in the anterior frontal cortex, larger defect on the left measuring 0.7 cm in keeping with open lip schizencephaly (B, C).

**Figure 3:**
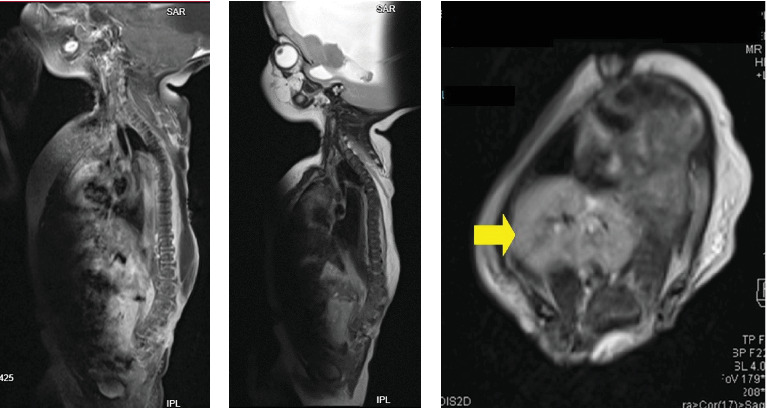
MRI spine and abdomen showing open defect in the back with absent posterior elements of the spine from the level of T4 with a short spinal cord seen ending at T6 level (A, B). The kidneys appear fused and seen in the right iliac region in location (C, yellow arrow). Features suggestive of severe hydrocephalus with corpus callosum agenesis and schizencephaly, spina bifida, and renal fusion.

MRI scan of the brain revealed severe dilatation of the lateral ventricles, with thinning of brain parenchyma, tectal beaking, and absent corpus callosum. Bilateral clefts in the anterior frontal cortex, larger defect on the left measuring 0.7 cm in keeping with open lip schizencephaly (Figure 2). The spine has an open defect in the back with absent posterior elements of the spine from the level of T4 with a short spinal cord seen ending at T6 level. The kidneys appear fused and are seen in the right iliac region in location. Features were suggestive of severe hydrocephalus with corpus callosum agenesis and schizencephaly, spina bifida, and renal fusion (Figure 3).

### 2.4. Therapeutic Intervention

**Figure 4:**
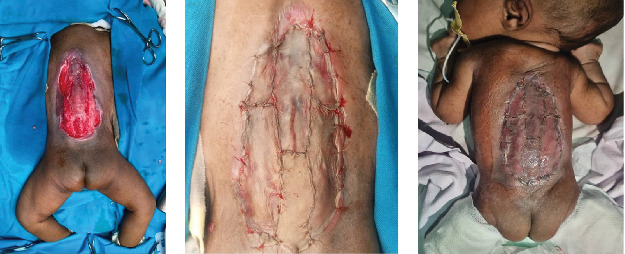
(A) Preoperative photograph, (B) meningomyelocele defect closed with skin grafting, and (C) 3 days postoperative.

After thorough counseling with the parents, the baby was planned for surgery for back closure of the meningomyelocele. This was done together with closure of the defect with partial thickness skin grafting (Figure 4), and ventriculoperitoneal shunting for the hydrocephalus.

### 2.5. Outcome and Follow-Up

The baby recovered well from anesthesia and had an uneventful postoperative course in the neonatal unit. Postoperative management included antibiotics and analgesia per local hospital guidelines, and initial feeding via nasogastric tube for 4 days, after which the baby transitioned to oral feeding. Discharged on day 20 postsurgery, the baby was reviewed 2 weeks later in the outpatient neurosurgery clinic. At follow-up, the surgical wounds were fully healed, with no signs of urine retention or seizures, and the mother reported successful breastfeeding. The occipital-frontal circumference was noted to be 42 cm, though no lower limb motor function was elicited. The child continues to be followed up by pediatricians, dietitians, and physiotherapists to ensure ongoing care and development.

## 3. Discussion

Spina bifida is a subset of neural tube defects occurring due to failure of complete fusion of the spinal neural tube. It consists of the splitting of the vertebral arches which may or may not involve the underlying neural tissue [4]. The most common areas of involvement include the lumbar and sacral spines followed by cervical and thoracic spine, but the involvement of multiple spinal segments is rare [5].

The global prevalence of neural tube defects is estimated at 4 per 1000 live births, of which in industrialized countries such as the United States and most European countries it ranges from 0.5 to 0.8 per 1000 live newborns each year. In Africa, the pooled birth prevalence of spina bifida is 0.13% with a range between 0.12% and 0.14%, the highest burden was detected in Algeria (0.43%), Ethiopia (0.32%), Tanzania (0.26%), Cameron (0.12%), Egypt (0.10%), and South Africa (0.10%) [3].

The etiology of spina bifida is usually multifactorial. Several potential risk factors including maternal alcohol intake, folate deficiency, smoking, gestational diabetes, valproic acid use, and environmental factors, such as organic solvents, nitrate-related compounds, pesticides, and polycyclic hydrocarbons are outlined [6]. Genetic factors including variations in some specific genes such as *MTHFR*, *MTHFD1*, *MTRR*, *VANGL1*, *VANGL2*, *CELSR1*, and *FUZ*, as well as variants in the T-locus on chromosome 6q have also been studied in the development of spina bifida and other spinal dysmorphisms [7].

Several other congenital abnormalities are associated with spina bifida, including hydrocephalus, Chiari 2 malformation, scoliosis, tethered cord, respiratory failure [8], and renal abnormalities including renal failure and renal fusion as seen in our case leading to earlier mortality in these cases [9].

Neural tube defects were first diagnosed prenatally in the 1970s with the discovery of elevated concentrations of alpha-fetoprotein in amniotic fluid samples from pregnancies with anencephaly or MMC. Later, acetylcholinesterase was also shown to be diagnostic. However, biochemical screening for MMC became unnecessary as ultrasounds offered greater sensitivity and specificity [10]. In our case, it became evident that the obstetric ultrasonography was performed by an inexperienced clinician, leading to the missed diagnosis of critical pathologies. This underscores the importance of educating the population on the value of early health-seeking behavior and the need for comprehensive training for radiology personnel, especially those working in primary care facilities. By enhancing their competence, such pathologies can be detected earlier, allowing timely referral to tertiary centers for specialized care.

Both ultrasound and MRI have significantly contributed to the diagnosis and management of spina bifida. Management strategies encompass both prenatal and postnatal approaches, including techniques such as prenatal fetoscopic open spina bifida repair, bilateral split latissimus dorsi V-Y flaps, single-stage back closure, and skin grafting [3, 11, 12]. In our patient's case, back closure for the defect was followed by ventriculoperitoneal shunt placement and skin grafting to cover the extensive skin defect.

Although the major aims of surgical closure are the removal of the malformed sac, the reestablishment of the normal CSF environment around the malformed spinal cord and thus restoring the normal functions of the placode, in addition to protecting it from infection by recreating the barrier with the exterior. All patients with spina bifida should be cared for with a multidisciplinary team involving neurosurgeons, pediatricians, neurologists, physiotherapists, and occupational therapists, to aid in preventing complications, monitoring, and managing other associated abnormalities [2].

## 4. Conclusion

Spina bifida involves a vast number of congenital spine defects with associated abnormalities leading to early mortality and life-long morbidity. Prenatal folic acid intake and other modifiable risk factors should be addressed early in maternal clinics together with early diagnosis and management.

## 5. Patient Perspective

The parents of the newborn were initially overwhelmed by the diagnosis of meningomyelocele, but they were grateful for the prompt and skilled surgical intervention their child received. The surgery was successful, and the parents continued to follow-up closely with the medical team. They remain hopeful for their child's future and are committed to ensuring the best possible care during the ongoing follow-up appointments.

## Data Availability

The authors have nothing to report.
